# Supplementary material for: Lack of a genetic cline and temporal genetic stability in an introduced barnacle along the Pacific coast of Japan
Source: PeerJ. 2022 Sep 28;10:e14073. doi: 10.7717/peerj.14073 (PMC9526406; doi:10.7717/peerj.14073)
Supplement: Supplemental Information 3 — (A) Average of daily max. and min. temperatures in Summer. (B) Average of daily max. and min. temperatures in Winter. (C) Linear regression between latitude of Balanus glandula sampling locations and daily average temperatures in August. (D) Linear regression between latitude of Balanus glandula sampling locations and daily average temperatures in February. Summer (August) amd Winter (February) temperature data of Japanese locations in 2018 were obtained from the website of Japan Meteorological Agency (https://www.jma.go.jp/jma/indexe.html, accessed on 2 August, 2022). Data of Summer (August or September) and Winter (January) temperatures in Southern (San Diego Lindbergh Fld), Central (Point Arena), North (Crescent City) California were obtained from the website of Western Regional Climate Center (https://wrcc.dri.edu/summary/Climsmnca.html, accessed on 2 August, 2022). [file peerj-10-14073-s003.docx]

Figure S1. Air temperatures of *Balanus glandula* sampling locations. (A) Average of daily max. and min. temperatures in Summer. (B) Average of daily max. and min. temperatures in Winter. (C) Linear regression between latitude of *Balanus glandula* sampling locations and daily average temperatures in August. (D) Linear regression between latitude of *Balanus glandula* sampling locations and daily average temperatures in February. Summer (August) amd Winter (February) temperature data of Japanese locations in 2018 were obtained from the website of Japan Meteorological Agency (https://www.jma.go.jp/jma/indexe.html, accessed on 2 Aug, 2022). Data of Summer (August or September) and Winter (January) temperatures in Southern (San Diego Lindbergh Fld), Central (Point Arena), North (Crescent City) California were obtained from the website of Western Regional Climate Center (https://wrcc.dri.edu/summary/Climsmnca.html, accessed on 2 Aug, 2022).
