# Supplementary material for: Lack of a genetic cline and temporal genetic stability in an introduced barnacle along the Pacific coast of Japan
Source: PeerJ. 2022 Sep 28;10:e14073. doi: 10.7717/peerj.14073 (PMC9526406; doi:10.7717/peerj.14073)
Supplement: Supplemental Information 4 — (A) Haplotype diversity at COI. (B) Nucleotide diversity at EF1. (C)Nucleotide diversity at COI. (B) Nucleotide diversity at EF1. Bars indicate standard deviation. [file peerj-10-14073-s004.docx]

Figure S2. Haplotype and nucleotide diversities of *Balanus glandula* at COI (607 bp) and EF1(292 bp). (A) Haplotype diversity at COI. (B) Nucleotide diversity at EF1. (C)Nucleotide diversity at COI. (B) Nucleotide diversity at EF1. Bars indicate standard deviation.
