# Supplementary material for: Lack of a genetic cline and temporal genetic stability in an introduced barnacle along the Pacific coast of Japan
Source: PeerJ. 2022 Sep 28;10:e14073. doi: 10.7717/peerj.14073 (PMC9526406; doi:10.7717/peerj.14073)
Supplement: Supplemental Information 5 — (A) Haplotype diversity at COI. (B) Nucleotide diversity at EF1. (C) Nucleotide diversity at COI. (B) Nucleotide diversity at EF1. Bars indicate standard deviation. [file peerj-10-14073-s005.docx]

Figure S3. Haplotype and nucleotide diversities of *Balanus glandula* across Japan at COI (386 bp) and EF1(154 bp) based on samples collected in this study (2019) and Geller et al (2008). (A) Haplotype diversity at COI. (B) Nucleotide diversity at EF1. (C) Nucleotide diversity at COI. (B) Nucleotide diversity at EF1. Bars indicate standard deviation.

**Reference**

Geller J, Sotka EE, Kado R, Palumbi SR, Schwindt E. 2008. Sources of invasions of a northeastern Pacific acorn barnacle, *Balanus glandula*, in Japan and Argentina. *Marine Ecology Progress Series* 358:211–218. DOI 10.3354/meps07466
